# Fetal monitoring from 39 weeks’ gestation to identify South Asian-born women at risk of perinatal compromise: a retrospective cohort study

**DOI:** 10.1038/s41598-021-02836-5

**Published:** 2021-12-02

**Authors:** Rebecca Stone, Kirsten Palmer, Euan M. Wallace, Mary-Ann Davey, Ryan Hodges, Miranda Davies-Tuck

**Affiliations:** 1 The Ritchie Centre, Hudson Institute of Medical Research, 27-31 Wright Street, Clayton, VIC 3168 Australia; 2 Department of Obstetrics and Gynaecology, Monash University, Clayton, VIC 3168 Australia; 3 Monash Health, 246 Clayton Road, Clayton, VIC 3168 Australia; 4 Safer Care Victoria, Melbourne, Australia; 5 Centre of Research Excellence in Stillbirth, Brisbane, Australia

**Keywords:** Risk factors, Diagnosis

## Abstract

To investigate whether earlier “post-term” monitoring of South Asian (SA) pregnancies from 39 weeks’ gestation with amniotic fluid index (AFI) and cardiotocography (CTG) detected suspected fetal compromise. Retrospective cohort study of all SA-born women at an Australian health service with uncomplicated, singleton pregnancies following the introduction of twice-weekly AFI and CTG monitoring from 39 weeks. Monitoring results, and their association with a perinatal compromise composite (including assisted delivery for fetal compromise, stillbirth, and NICU admission) were determined. 771 SA-born women had earlier monitoring, triggering delivery in 82 (10.6%). 31 (4%) had a non-reassuring antepartum CTG (abnormal fetal heart rate or variability, or decelerations) and 21 (2.7%) had an abnormal AFI (≤ 5 cm). Women with abnormal monitoring were 53% (95% CI 1.2–1.9) more likely to experience perinatal compromise and 83% (95% CI 1.2–2.9) more likely to experience intrapartum compromise than women with normal monitoring. Monitoring from 39 weeks identified possible fetal compromise earlier than it otherwise would have been, and triggered intervention in 10% of women. Without robust evidence to guide timing of birth in SA-born women to reduce rates of stillbirth, earlier monitoring provides an alternative to routine induction of labour.

## Introduction

Women of South Asian (SA) background (e.g. those born on India, Sri Lanka, Pakistan and Bangladesh) experience higher rates of stillbirth at term than other women^1–6^. The mechanism underlying this increased risk is thought to be earlier maturation of the placenta in South Asian women^7,8^. Not only is the rate of stillbirth among SA women at term higher than for other women, but the rate increases earlier in pregnancy and more rapidly, such that SA women have a rate of stillbirth at 39 weeks’ gestation similar to Australian women at 41 weeks^1^. Current clinical practice guidelines on care of post-term pregnancies recommend routine ultrasound assessment and/or induction of pregnancies at 41 weeks’, agnostic to maternal ethnicity^9,10^. By the time SA women are offered monitoring of the wellbeing of their baby, they are already at between 2 and 5 times the risk of stillbirth compared to the Australian-born population^1,2,11^.

In recognition of this, in mid-2017 a large Victorian maternity service implemented a new clinical practice guideline for “post-term” care of South Asian women. One-third of all women giving birth at Monash Health are of SA background (~ 3000 per year). With a simple change to “traditional” post-term care guidelines, women of SA background are offered antepartum fetal monitoring with cardiotocography (CTG) and amniotic fluid index (AFI) measurement twice weekly from 39 weeks’ gestation, rather than 41 weeks’ gestation as it is for other women. We hypothesised that earlier monitoring of SA women would improve the detection of babies at risk of stillbirth, and other adverse perinatal outcomes.

This study aimed to illustrate who underwent fetal monitoring, the rates of abnormal monitoring detected, and whether earlier antepartum monitoring in South Asian women predicts adverse perinatal outcomes.

## Methods

### Study design

Retrospective observational study at Monash Health, Victoria’s largest metropolitan health service caring for ~ 10,000 pregnancies per year across three separate hospital sites of different acuity levels.

**Figure 1:**
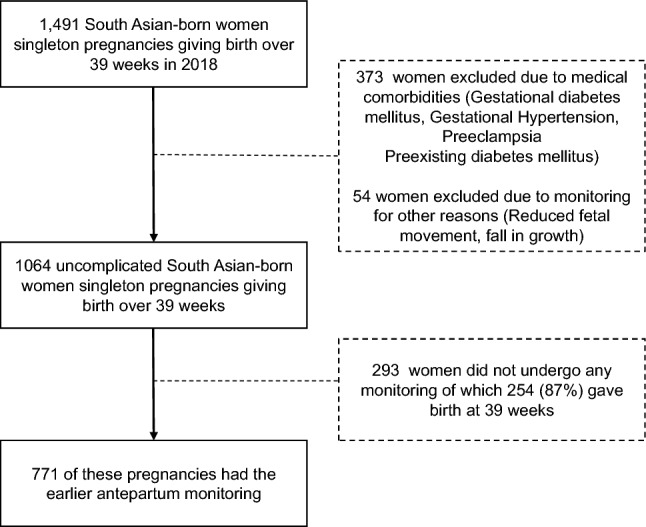
Flow chart of singleton pregnancies of South Asian-born women at Monash Health in 2018 and those who underwent earlier “post-term” antepartum monitoring from 39 weeks.

Mothers born in South Asia giving birth ≥ 39 weeks’ gestation at Monash Health in 2018 were included in this study. Maternal country of birth was used as a surrogate for ethnicity. Women who reported being born in Afghanistan, Bangladesh, Bhutan, India, Iran, the Maldives, Nepal, Pakistan, or Sri Lanka^12^ were included. Women were excluded if they had a multiple pregnancy, gave birth prior to 39 weeks’ gestation, had maternal medical conditions including gestational diabetes, preeclampsia, or gestational hypertension or if they underwent monitoring for other reasons. A flow chart of the exclusions is provided in Fig. 1. All women were offered earlier “post-term” monitoring from 39 weeks under the new clinical guideline that recommended twice weekly fetal surveillance with cardiotocography (CTG) and measurement of amniotic fluid from 39 weeks’ gestation for women born in South Asia. Induction of labour (IOL) was offered from 41 weeks as it remains for all other women.

Data were extracted from the Birthing Outcomes System (BOS), Monash Health’s Scanned Medical Records (SMR) and Vue Motion Imaging.

The primary exposures were antepartum CTG and AFI results of women having earlier post-term monitoring under the guideline. Due to appointment scheduling some antepartum monitoring was undertaken from 38 weeks’ gestation and included in this study. The CTGs were categorised into normal and non-reassuring according to national guidelines^13,14^. Normal CTGs met the following criteria: baseline rate 110–160 bpm, baseline variability of 6–25 bpm, accelerations 15 bpm for 15 s, no decelerations^15^. If this criteria was not met, the CTG was categorised as non-reassuring.

AFIs were reported as raw values and categorised into 0–5 cm, > 5–8 cm, 9–14 cm, 15–20 cm, 20–25 cm and > 25 cm at each monitoring episode and for each gestational week. If women had two episodes of monitoring in the same gestational week, the lowest AFI was reported. We created a variable for an abnormal CTG at any monitoring and oligohydramnios 0–5 cm, borderline (> 5–8 cm) or polyhydramnios (> 25 cm) at monitoring prior to 41 weeks’ gestation to capture abnormalities detected with the earlier monitoring. Women who moved between groups were classified in the lowest AFI group.

The primary outcome was a composite of intrapartum fetal and/or neonatal compromise. Intrapartum compromise was defined as an abnormal intrapartum CTG or fetal heart rate through either continuous electronic fetal monitoring or intermittent auscultation triggering intervention through instrumental delivery or caesarean section, abnormal fetal blood sampling during labour (lactate > 4.8 mmol/L or pH < 7.20), or stillbirth as defined using standard clinical definitions^13,16^. Neonatal compromise was defined as neonatal death, five-minute Apgar scores < 7, resuscitation with either continuous positive airway pressure (CPAP), intermittent positive pressure respiration (IPPR), or intubation, cord lactate levels > 6.8 mmol/L, or special care nursery/NICU admission. The above outcomes were identified through review of mothers’ and babies’ medical records.

Secondary outcomes included whether antepartum monitoring triggered interventions. This was collected as onset of labour (spontaneous, induced, or no labour), indications for induction, instrumental birth or caesarean sections (including abnormal AFI, abnormal CTG) and mode of birth (spontaneous vaginal, instrumental, or caesarean).

Maternal age, body mass index, parity, smoking status, gestation at birth, baby sex and birthweight were also extracted.

### Statistical analysis

Continuous variables were assessed for normality. Baseline characteristics, obstetric interventions and rates of primary and secondary outcomes were compared between those with and without earlier monitoring using standard approaches. The primary and secondary outcomes as well as the individual components of the composite were compared between those with and those without abnormal monitoring. Poisson regression was then used to calculate incidence rate ratios for the primary and secondary composite outcomes according to abnormal monitoring overall and for those with an abnormal CTG or oligohydramnios identified prior to 41 weeks’ gestation, adjusting for the potential prespecified confounders age, body mass index, parity and gestation at birth to determine the association between abnormalities detected through the earlier monitoring and compromise. CTG and oligohydramnios were also included with they were considered separately. Episodes of monitoring and subsequent results and birth outcomes were also described. Stata 12.0 (StataCorp, USA). A p value < 0.05 was considered statistically significant.

Ethics approval for this project was obtained from the Monash Health Human Research Ethics Committee under the Monash Health reference of RES-18-0000-186L (HREC/18/MonH/171), which contained a waiver on the need to obtain individual informed consent given the study used only retrospective, routinely-collected data.

### Ethics

Ethics approval for this project was obtained from the Monash Health Human Research Ethics Committee under the Monash Health reference of RES-18-0000-186L (HREC/18/MonH/171). The study was performed in accordance with the Declaration of Helsinki.

## Results

1064 South Asian-born women with uncomplicated pregnancies gave birth in 2018 at 39 weeks’ gestation or more. Antepartum monitoring ≥ 39 weeks’ gestation was performed in 771 of these women (73%) and 293 (27%) women did not receive fetal monitoring. Women who did not undergo any monitoring were more likely to be older, parous, give birth at 39 weeks, have lighter babies and give birth by planned elective caesarean section. Overall, the rates of the primary composite (38.1% vs 29%; p = 0.006) and intrapartum composite (18% vs 13%; p = 0.03) were higher in women who underwent post-term monitoring compared to those who had no monitoring (Table 1).

**Table 1:** Characteristics of South Asian-born women with uncomplicated singleton pregnancies without and with antepartum monitoring.

	No earlier monitoring n = 293	Earlier monitoring n = 771	p value
Maternal age			**0.03**
≤ 20	2 (0.7)	12 (1.6)	
> 20–34.9	234 (79.9)	656 (85)	
35–39.9	47 (16)	92 (12)	
≥ 40	10 (3.4)	11 (1.4)	
Maternal BMI^#^	25 (4.2)	24.9 (3.8)	0.74
Parous	213 (72.7)	435 (56.2)	** < 0.001**
Smoker	1 (0.3)	4 (0.5)	0.71
Birth weight (g)^#^	3365 (400)	3492 (421)	** < 0.001**
Gestation at birth in weeks			** < 0.001**
39	254 (87)	259 (33.6)	
40	32 (10.9)	384 (49.8)	
41	6 (2.1)	122 (15.8)	
42	1 (0.3)	6 (0.8)	
Onset of labour			** < 0.001**
Spontaneous	120 (41))	404 (52)	
Induced	79 (27)	317 (41)	
No labour	94 (32)	50 (6.5)	
Mode of birth			** < 0.001**
Normal vaginal	127 (43.3)	424 (55)	
Instrumental	41 (14)	173 (22.4)	
Elective caesarean	89 (30.4)	40 (5.2)	
Emergency caesarean	35 (12)	134 (17.4)	
Vaginal breech	1 (0.3)	0	
Stillbirths	0 (0)	1 (0.13)	0.54
Primary composite outcome	85 (29)	294 (38.1)	**0.006**
Intrapartum composite outcome	39 (13.3)	145 (18.8)	**0.03**
Neonatal composite outcome	63 (21.5)	208 (27)	0.07

Number of (%) unless otherwise stated. ^#^Mean (SD). Significant values in bold.

**Figure 2:**
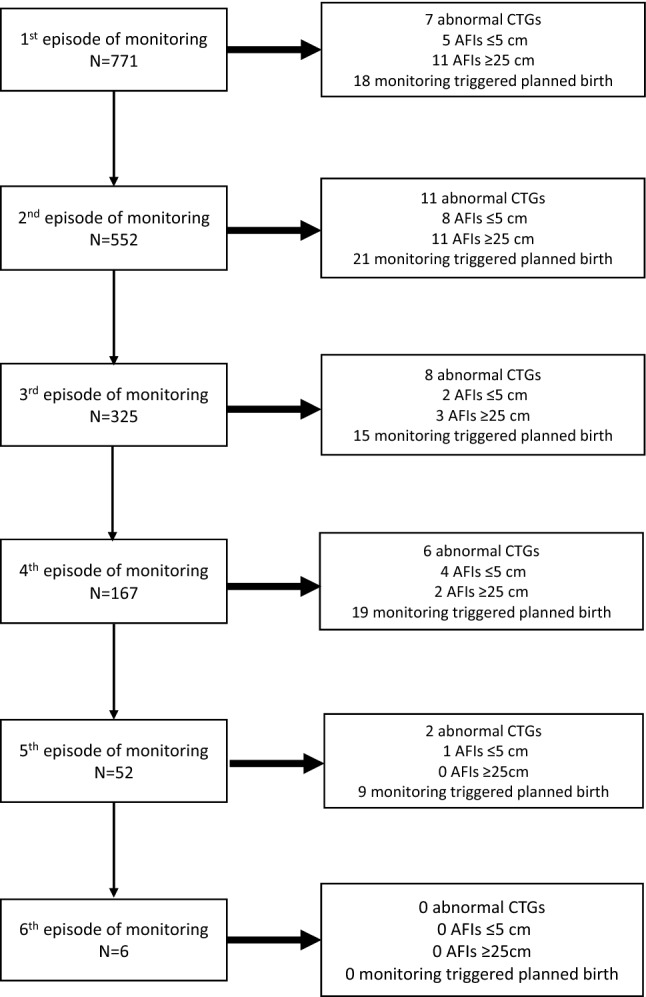
Monitoring experiences of South Asian-born women with uncomplicated, singleton pregnancies in 2018 with “post-term” monitoring from 39 weeks.

Among the women who underwent earlier “post-term” monitoring as per the clinical guideline, on average these women underwent two episodes of monitoring (median 2, IQR 1–3) before giving birth. An abnormal antepartum CTG was reported in 31 women (4%). All abnormal CTGs were detected prior to 41 weeks. A total of 21 women (2.7%) had an AFI ≤ 5 cm at any point, of which 20 (95%) were identified prior to 41 weeks’ gestation and 13 (62%) prior to 40 weeks. 16 women had an AFI > 25 cm at any point of which 14 (88%) were identified prior to 40 weeks. Monitoring results from earlier “post-term” monitoring triggered earlier delivery in 82 (10.6%) women (67 inductions, 11 caesarean births). The trigger for intervention included the results of that monitoring episode or reflected multiple monitoring episodes (e.g. fall in AFI, or more than one low measure). Four women who had intervention triggered went into spontaneous labour prior to the planned birth. Overall, 37.8% (n = 31) of women in whom intervention was triggered gave birth at 39 weeks’ gestation, 50% (n = 41) gave birth at 40 weeks and 12.2% (n = 10) gave birth at 41 weeks. The flowchart of women through the monitoring journey is presented in Fig. 2.

The results of the monitoring were abnormal for 47 (6%) of women. Just over half (54.8%) of women who had abnormal monitoring experienced perinatal compromise compared to 37% of women whose morning was normal. The time between the first monitoring episode and birth was not significantly different between women with and without abnormal monitoring (1.0 (SD0.02) vs 0.88 (SD 0.8 weeks; p = 0.13) however women who had abnormal monitoring gave birth at an earlier gestation (39.6 vs 39.9 weeks; p = 0.03). Abnormal monitoring was associated with a 53% (95%CI 1.20–1.90) increased risk of perinatal compromise and a 83% (95% CI 1.2–2.9) increased risk of intrapartum compromise after adjusting for potential confounders. No increased risk of neonatal compromise was observed. When the individual components of the abnormal monitoring were examined an abnormal CTG was associated with both increased risk of perinatal and intrapartum but not neonatal compromise. Oligohydramnios was not significantly associated with either perinatal, intrapartum or neonatal compromise (Table 2).

**Table 2:** Individual components of the composite outcomes.

	Normal monitoring n = 724	Abnormal monitoring N = 47	P value
Stillbirth	0 (0)	1 (2.1)	** < 0.001**
Neonatal death	0 (0)	1 (2.1)	** < 0.001**
SCN/NICU admission	103 (14)	10 (21)	0.19
5 min Apgar < 7	9 (1.2)	2 (4.3)	0.09
Fetal blood sampling < 4.8	2 (0.3)	0 (0)	0.72
Cord lactate > 6.8	63 (8.7)	2 (4.3)	0.29
Resuscitation	72 (9.9)	6 (13)	0.53
Assisted birth for suspected fetal compromise	129 (17.8)	16 (34)	**0.006**

Some women experienced more than one complication.

Data is presented as number (%). Significant values in bold.

The individual components of the composite perinatal compromise outcome for women with and without abnormal monitoring are presented in Table 3. Women who had abnormal monitoring were significantly more likely to experience perinatal death and an assisted delivery due to fetal compromise. While not statistically significant the rates of SCN/NICU admission, 5 min Apgar < 7 and need for resuscitation were higher in women with abnormal monitoring.

**Table 3:** Rates of primary outcomes by monitoring results and components of monitoring results.

	Normal monitoring n = 724	Abnormal monitoring N = 47	p value	Adjusted RR (95% CI)	p value
Primary composite outcome	267 (37)	27 (54.8)	**0.005**	1.53 (1.2–1.9)	**0.001**
Intrapartum composite	129 (18)	16 (34)	**0.006**	1.83 (1.2–2.9)	**0.007**
Neonatal composite	193 (27)	15 (32)	0.43	1.20 (0.79–1.82)	0.40
	**Normal CTG N = 737**	**Abnormal CTG N = 31**			
Primary composite outcome	273 (37)	19 (61)	**0.006**	1.53 (1.15–2.0)	**0.003**
Intrapartum composite	133 (18)	12 (39)	**0.004**	1.53 (1.2–2.0)	**0.003**
Neonatal composite	195 (26.5)	11 (35)	0.27	1.29 (0.8–2.1)	0.30
	**Normal AFI N = 749**	**Oligohydramnios N = 21**			
Primary composite outcome	282 (38)	11 (52)	0.17	1.32 (0.91–1.91)	0.14
Intrapartum composite	139 (19)	6 (28.6)	0.54	1.3 (0.9–1.9)	0.14
Neonatal composite	202 (27)	4 (22.2)	0.87	1.07 (0.57–2.0)	0.83

Adjusted for age, body mass index, parity and gestation at birth. AFI/CTG also included when CTG and Oligohydramnios were compared separately. Significant values in bold.

## Discussion

We found that earlier “post-term” monitoring in South Asian-born women identified abnormal CTGs and AFI findings that may not have otherwise been detected. Findings from the monitoring triggered earlier delivery in 10.6% of women and abnormal “earlier fetal monitoring” was associated with a higher rate of intrapartum and combined intrapartum and neonatal compromise. This was driven by the abnormal CTG, not identification of oligohydramnios.

Typical “post-term” monitoring in Australia involves fetal surveillance from 41 weeks’ gestation while awaiting spontaneous labour to begin due to the elevated risk of perinatal death in the post term period. We have previously shown that SA-born women have increased rates of abnormal “traditional post-term” monitoring from 41 weeks’ gestation^17^, increased rates of intrapartum compromise at term^18^, and are more likely to experience stillbirth at term, with rates at 39 weeks equivalent to other women at 41 weeks^1^. In this study we found that all abnormal CTGs and the majority of abnormal AFIs in South Asian-born women undergoing earlier “post-term” surveillance were detected between prior to 41 weeks’ gestation. Waiting until 41 weeks’ gestation, as per current guidelines for prolonged pregnancy management may therefore be too late in this group of women.

Just under 40% of the otherwise healthy SA-born women experienced perinatal compromise, and abnormal fetal monitoring was associated with perinatal compromise overall and the risk of intrapartum but not neonatal compromise. The most common type of compromise that occurred was assisted birth due to fetal compromise. We have previously reported that ~ 42–49% of South Asian born women at term experience an assisted birth due to fetal compromise^19^. In this current study 18.8% of South Asian born women overall term experienced an assisted birth due to fetal compromise, and 34% of those with abnormal monitoring did. Detection and birth of the potentially compromised neonate earlier than it otherwise may have may have resulted in neonates being better able to cope with labour and aligns with the reduced rates of caesarean births that were observed in the ARRIVE trial which compared low risk women undergoing IOL at 39 weeks to those with to expectant management^20^. When the components of the monitoring were examined separately, only abnormal CTG was associated with compromise. These findings were not surprising given the poor sensitivity and predictive values of amniotic liquor volume (AFI) to assess fetal wellbeing^21,22^. The addition of other components, including fetoplacental Dopplers, and measurement of biomarkers in maternal blood, such as placental growth factor (PlGF) and soluble fms-like tyrosine kinase-1 (sFLT-1) may improve the ability of surveillance to identify at-risk fetuses^23^ with further research needed.

An alternative approach to reduce stillbirth in South Asian-born women would be simply to offer routine IOL. Findings from a Canadian RCT of women randomised to IOL or fetal monitoring did not observe a significant difference in rates of stillbirth for women undergoing monitoring^24^. In this study IOL and monitoring however was commenced at 41 weeks. While there is good evidence that IOL at 41 weeks’ gestation reduces the rate of stillbirth^25^, there remains debate arounds IOL at earlier gestations at term^26^. Recent findings from the ARRIVE trial also did not show a significant reduction in perinatal death^20^. Future research is needed. Regardless, even in women at high risk of stillbirth, such as women of South Asian ethnicity, over 3500 women would need to be induced to prevent one stillbirth^27^. In the normal “low risk” pregnant population the “number needed to treat” increases to nearly 40,000 women^27^. There is also concern about the short- and long-term adverse effects of iatrogenic early term births. Being born at 37–39 weeks’ gestation, as opposed to > 40 weeks, is associated with a higher rate of immediate perinatal morbidity, hospitalisation in the first five years of life, childhood diabetes, and impaired primary school performance^28,29^. Whether earlier birth in SA-born women reduces adverse outcomes without causing harm is unknown. The findings of our study suggest earlier fetal surveillance may offer an alternative to routine earlier IOL for women of South Asian ethnicity. Earlier fetal monitoring identified and subsequently triggered the earlier delivery of 1 in 10 women. Furthermore it also allowed 52% of women at term to go into spontaneous labour.

Our study has a number of limitations. Firstly, the primary outcome is a composite variable as the sample size was too small to provide meaningful insight into rare outcomes such as stillbirth. As maternal ethnicity is not routinely recorded in medical records, region of birth was used as a surrogate measure. Our study likely misses out on women of South Asian ethnicity born in regions outside of South Asia. As a retrospective observational study, this study relied on accuracy of data reporting, which cannot be guaranteed. Both CTG and AFI have a degree of variability in user interpretation, likely exacerbated by the retrospective nature of this study.

In conclusion, this study shows that earlier monitoring from 39 weeks’ gestation in SA-born women with twice weekly AFI and CTG identified possible fetal compromise earlier in gestation than it otherwise would have been and triggered intervention in 10% of women. Adverse outcomes were only associated with an abnormal CTG. Restricting earlier fetal monitoring to CTG may be sufficient to identify the baby at risk, minimise likelihood of a false positive and reduce the costs to the health service. In the absence of good evidence to guide timing of birth in South Asian born women to reduce the rates of stillbirth, fetal monitoring provides an alternative to routine earlier IOL.
